# Proposed New Strain of Canine *Kobuvirus* from Fecal Samples of Brazilian Domestic Dogs

**DOI:** 10.1128/MRA.01292-18

**Published:** 2019-01-03

**Authors:** Bruno de Cássio Veloso Barros, Ceyla Maria Oeiras Castro, Diego Pereira, Laila Graziela Ribeiro, José Wandilson Barboza Duarte Júnior, Samir Mansour Moraes Casseb, Gustavo Moraes Holanda, Ana Cecília Ribeiro Cruz, Edivaldo Costa Sousa Júnior, Joana D’Arc Pereira Mascarenhas

**Affiliations:** a Section of Virology, Evandro Chagas Institute, Ministry of Health, Ananindeua, Brazil; b Section of Arbovirology and Hemorrhagic Fevers, Evandro Chagas Institute, Ministry of Health, Ananindeua, Brazil; University of Arizona

## Abstract

A proposed new strain of canine *Kobuvirus* was identified in fecal samples of domestic dogs from a rural community located in the municipality of Peixe-Boi, Pará, Brazil. The nucleotide identity was 92.3% similar to other representatives of the family *Picornaviridae*, genus *Kobuvirus*, and species *Aichivirus A*, which suggests that this is possibly a new strain within this species.

## ANNOUNCEMENT

*Kobuvirus* (KoV) belongs to the *Picornaviridae* family, which includes pathogens that infect humans and animals. Since the discovery of the first KoV in a patient with gastroenteritis in March 1989 in Japan (1), other strains have been identified as members of the genus *Kobuvirus* from a variety of domestic and wild animal species, including canine, feline, murine, avian, ovine, bovine, musteline, caprine, and porcine species and wild boars in several regions of the world (2). KoVs are icosahedral and nonenveloped particles which present a genomic organization similar to that of other members of the *Picornaviridae* family and have a linear and positive-sense single-stranded RNA (ssRNA) genome of 8.2 to 8.4 kb, containing a large open reading frame (ORF) coding a single-polyprotein precursor and a 5′ and a 3′ untranslated region (UTR). The polyprotein generates three structural proteins and eight nonstructural proteins (3). In October 2016, 35 fecal samples of nondiarrheic dogs, aged between 5 months and 8 years, were collected at the same time from a rural community located in the municipality of Peixe-Boi, Pará, Brazil.

A cDNA library was prepared and sequenced on an Illumina MiniSeq platform with the methodology described in the Nextera XT DNA library preparation kit (4) with 150-bp paired-end reads. The genome was assembled using a hybrid methodology of *de novo* assembly and reference mapping with the Minia program (kmer size, 32; abndance-min, 3; abundance-max, 500; nb-cores, 8) (5) and the Geneious (default parameters) version 8.1.9 (6) program, respectively. The taxonomic annotation was performed with Kraken software (default parameters) (7). The sequencing produced 3,619,638 reads, and 22,224 contigs were generated, 52 of which were related to the *Picornaviridae* family. The resulting sequence was subsequently characterized as *Canine Kobuvirus* 26 (CaKoV-26) after sequence editing, which was performed on ambiguous bases, and the unitig was inspected and compared with the reference sequence. The genome showed a final length of 8,312 bp. The assembly with reference mapping showed that 390 reads generated a single unitig related to the canine *Kobuvirus* CH-1 strain, species *Aichivirus A* (GenBank accession no. JQ911763), which was the closest sequence using Kraken, presenting 92.3% nucleotide identity with a new strain, CaKov-26. In the P1, P2, and P3 polyprotein regions, the nucleotide identities were 89.6%, 95.9%, and 94.9%, and the amino acid identities were 94%, 99.8%, and 98.7%, respectively. The average coverage of reads was 7.1×, and the GC content was 58.1%. The average similarity between the different KoVs was 92% to 98%, and the CaKoV-26 strain showed a common identity that is related to these viruses. The ORF search to define the polyprotein region, domains, and subdomains was annotated by comparison with 59 other *Picornaviridae* genome sequences.

Several studies in Brazil have demonstrated the circulation of KoV strains in different host species and geographical locations (8–14), but herein, we describe the first complete genome sequence of the new canine *Kobuvirus* strain in nondiarrheic dogs and highlight the importance of KoV in the context of infections. Since there are few studies about this virus, we aim to contribute to a better understanding of its epidemiological, pathogenic, and zoonotic aspects.

### Data availability.

The complete genome sequence of canine *Kobuvirus* strain CaKoV-26 was deposited in GenBank under the accession no. MH747478.

## References

[1] Reuter G, Boros Á, Pankovics P 2011 Kobuviruses—a comprehensive review. Rev Med Virol 21:32–41. doi:10.1002/rmv.677. 21294214

[2] Pankovics P, Boros Á, Bíró H, Horváth KB, Phan TG, Delwart E, Reuter G 2016 Novel picornavirus in domestic rabbits (*Oryctolagus cuniculus* var. *domestica*). Infect Genet Evol 37:117–122. doi:10.1016/j.meegid.2015.11.012. 26588888 PMC7172602

[3] Lu G, Zhang X, Luo J, Sun Y, Xu H, Huang J, Ou J, Li S 2018 First report and genetic characterization of feline kobuvirus in diarrhoeic cats in China. Transbound Emerg Dis 65:1357–1363. doi:10.1111/tbed.12916. 29873199 PMC7169872

[4] Zhong S, Joung J-G, Zheng Y, Chen Y-R, Liu B, Shao Y, Xiang JZ, Fei Z, Giovannoni JJ 2011 High-throughput Illumina strand-speciﬁc RNA sequencing library preparation. Cold Spring Harb Protoc 2011:940–949. doi:10.1101/pdb.prot5652. 21807852

[5] Chikhi R, Rizk G 2013 Space-efficient and exact de Bruijn graph representation based on a Bloom filter. Algorithms Mol Biol 8:22. doi:10.1186/1748-7188-8-22. 24040893 PMC3848682

[6] Kearse M, Moir R, Wilson A, Stones-Havas S, Cheung M, Sturrock S, Buxton S, Cooper A, Markowitz S, Duran C, Thierer T, Ashton B, Meintjes P, Drummond A 2012 Geneious Basic: an integrated and extendable desktop software platform for the organization and analysis of sequence data. Bioinformatics 28:1647–1649. doi:10.1093/bioinformatics/bts199. 22543367 PMC3371832

[7] Wood DE, Steven LS 2014 Kraken: ultrafast metagenomic sequence classification using exact alignments. Genome Biol 15:R46. doi:10.1186/gb-2014-15-3-r46. 24580807 PMC4053813

[8] Barry AF, Ribeiro J, Alfieri AF, van der Poel WHM, Alfieri AA 2011 First detection of kobuvirus in farm animals in Brazil and the Netherlands. Infect Genet Evol 11:1811–1814. doi:10.1016/j.meegid.2011.06.020. 21763785

[9] Ribeiro J, de Arruda Leme R, Alfieri AF, Alfieri AA 2013 High frequency of *Aichivirus C* (porcine kobuvirus) infection in piglets from different geographic regions of Brazil. Trop Anim Health Prod 45:1757–1762. doi:10.1007/s11250-013-0428-x. 23765550

[10] Ribeiro J, Lorenzetti E, Alfieri AF, Alfieri AA 2014 Kobuvirus (*Aichivirus B*) infection in Brazilian cattle herds. Vet Res Commun 38:177–182. doi:10.1007/s11259-014-9600-7. 24590582 PMC7088743

[11] Ribeiro J, Headley SA, Diniz JA, Pereira AHT, Lorenzetti E, Alfieri AA, Alfieri AF 2017 Extra-intestinal detection of canine kobuvirus in a puppy from southern Brazil. Arch Virol 162:867–872. doi:10.1007/s00705-016-3164-5. 27888408 PMC7086620

[12] Ribeiro J, Lorenzetti E, Júnior JCR, da Silva Medeiros TN, Alfieri AF, Alfieri AA 2017 Phylogenetic analysis of VP1 and RdRP genes of Brazilian aichivirus B strains involved in a diarrhea outbreak in dairy calves. Arch Virol 162:3691–3696. doi:10.1007/s00705-017-3531-x. 28849283 PMC7086745

[13] Portes SAR, Volotao EDM, Rose TL, Roch MS, Xavier MDPTP, de Assis RM, Fialho AM, Rocha MS, Miagostovich MP, Liete JPG, Carvalho-Co FA 2015 Aichi virus positivity in HIV-1 seropositive children hospitalized with diarrheal disease. Curr HIV Res 13:325–331. doi:10.2174/1570162X13666150511145950. 26081831

[14] Candido M, Batinga MCA, Alencar ALF, de Almeida-Queiroz SR, Buzinaro MDG, Livonesi MC, Fernandes AM, de Sousa RLM 2017 Molecular characterization and genetic diversity of bovine *Kobuvirus*, Brazil. Virus Genes 53:105–110. doi:10.1007/s11262-016-1391-1. 27623839

